# GSK3B induces autophagy by phosphorylating ULK1

**DOI:** 10.1038/s12276-021-00570-6

**Published:** 2021-03-02

**Authors:** Hye Young Ryu, Leah Eunjung Kim, Hyeonjeong Jeong, Bo Kyoung Yeo, Ji-Won Lee, Hyeri Nam, Shinwon Ha, Hyun-Kyu An, Hyunhee Park, Seonghee Jung, Kyung Min Chung, Jiyea Kim, Byung-Hoon Lee, Heesun Cheong, Eun-Kyoung Kim, Seong-Woon Yu

**Affiliations:** 1 Department of Brain and Cognitive Sciences, Daegu Gyeongbuk Institute of Science and Technology (DGIST), Daegu, Republic of Korea; 2 Division of Cancer Biology, Research Institute, National Cancer Center, 10408 Goyang, Republic of Korea; 3 Department of New Biology, Daegu Gyeongbuk Institute of Science and Technology (DGIST), Daegu, Republic of Korea; 4 Neurometabolomics Research Center, Daegu Gyeongbuk Institute of Science and Technology (DGIST), Daegu, Republic of Korea

**Keywords:** Macroautophagy, Phosphorylation

## Abstract

Unc-51-like autophagy activating kinase 1 (ULK1), a mammalian homolog of the yeast kinase Atg1, has an essential role in autophagy induction. In nutrient and growth factor signaling, ULK1 activity is regulated by various posttranslational modifications, including phosphorylation, acetylation, and ubiquitination. We previously identified glycogen synthase kinase 3 beta (GSK3B) as an upstream regulator of insulin withdrawal-induced autophagy in adult hippocampal neural stem cells. Here, we report that following insulin withdrawal, GSK3B directly interacted with and activated ULK1 via phosphorylation of S405 and S415 within the GABARAP-interacting region. Phosphorylation of these residues facilitated the interaction of ULK1 with MAP1LC3B and GABARAPL1, while phosphorylation-defective mutants of ULK1 failed to do so and could not induce autophagy flux. Furthermore, high phosphorylation levels of ULK1 at S405 and S415 were observed in human pancreatic cancer cell lines, all of which are known to exhibit high levels of autophagy. Our results reveal the importance of GSK3B-mediated phosphorylation for ULK1 regulation and autophagy induction and potentially for tumorigenesis.

## Cell biology: Modified enzyme implicated in tumor formation

Similar to cellular starvation conditions, insulin withdrawal may trigger the modification of an enzyme involved in the induction of autophagy, a key cellular recycling process. The ULK1 enzyme has a critical role in autophagy induction. Seong-Woon Yu at the Daegu Gyeongbuk Institute of Science and Technology, South Korea, and co-workers investigated how ULK1 is activated under insulin withdrawal condition. They found that another enzyme called GSK3B modifies two specific ULK1 amino acids, activating ULK1 and triggering autophagy. Further, they found high levels of this type of ULK1 modification in human pancreatic cancer cell lines that exhibited increased autophagy, suggesting possible implications for the development of certain cancerous tumors.

## Introduction

Macroautophagy (hereafter referred to as autophagy) is an evolutionarily conserved intracellular catabolic process with various context-dependent functions in cell survival or death^1^. Constitutive autophagy at the basal level is pivotal for protein quality control and normal growth via the turnover of intracellular macromolecules and organelles. Autophagy can also be induced under a variety of stress conditions, such as nutrient or growth factor depletion, hypoxia, and pathogen infection^2^. Autophagy is characterized by an increase in the formation of double-membrane autophagosomes, which are mediated by autophagy-related (*Atg*) genes. Our knowledge of the molecular mechanisms and genes involved in autophagy has greatly advanced since the initial discovery of these genes in yeast and later in various organisms, including humans^3–6^.

Yeast Atg1 and its mammalian homolog, unc-51-like autophagy activating kinase 1 (ULK1), are serine/threonine protein kinases that initiate autophagosome formation^7^. The ULK family includes five homologs, ULK1, ULK2, ULK3, ULK4, and STK36^8^. Among them, ULK1 and ULK2 have been primarily studied for their roles in autophagy induction^8^. ULK1 and ULK2 share an overall 52% amino acid identity^9^. However, despite this sequence identity, ULK1 appears to be more important than ULK2 for autophagy induction upon nutrient starvation^10^, although some studies have reported that deletion of both the *Ulk1* and *Ulk2* genes is required to block starvation-induced autophagy^10–13^. ULK1 forms a tetrameric complex, the so-called “initiator complex” containing ATG13, RB1-inducible coiled-coil 1 (RB1CC1/FIP200), and ATG101^14,15^.

Due to its pivotal role in autophagy initiation, much effort has been concentrated on revealing the mechanisms regulating ULK1 activation. During autophagosome assembly, the formation of the ULK1–ATG13–RB1CC1–ATG101 complex is regulated by several nutrient status-sensing molecules, including a mechanistic target of rapamycin complex 1 (MTORC1) and AMP-activated protein kinase (AMPK)^16^. Under fed conditions, MTORC1 directly interacts with ULK1 via its subunit regulatory associated protein of MTOR complex 1 and phosphorylates ULK1 at multiple sites, including S757, to repress ULK1 kinase activity^17,18^. However, upon nutrient starvation or rapamycin treatment, MTORC1 dissociates from the ULK1 complex, which results in hypophosphorylation of ULK1 and ATG13. Then, ULK1 is activated by autophosphorylation and phosphorylates both ATG13 and RB1CC1, leading to translocation of the initiator complex to a phagophore, a cup-shaped precursor of the autophagosome^10,18^. AMPK is a cellular energy sensor that regulates cell metabolism and autophagy^19,20^. Several lines of evidence have suggested that AMPK positively regulates autophagy via phosphorylation of ULK1 at multiple sites, including S317 and S777, under starvation conditions^16^.

The autophagosome formation process consists of nucleation, elongation, and the closure and maturation of the autophagosome membrane, and these steps require two ubiquitin-like protein conjugation systems. One system involves ATG12, which is conjugated to ATG5, and their dimer binds ATG16L1 to form the ATG12–ATG5–ATG16L1 complex^21,22^. The other system involves ATG8^23,24^. Unlike yeast, which has a single Atg8 protein, the mammalian ATG8 protein family includes at least 7 members in two subfamilies: the microtubule-associated protein 1 light chain 3 (MAP1LC3) subfamily, which includes MAP1LC3A, MAP1LC3B, MAP1LC3B2, and MAP1LC3C, and the gamma-aminobutyric acid type A receptor-associated protein (GABARAP) subfamily, which includes GABARAP, GABARAP-like 1 (GABARAPL1), and GABARAP-like 2 (GABARAPL2)^25^. Among them, the best-studied protein is MAP1LC3B^26,27^. Upon translation, MAP1LC3B is cleaved by ATG4 protease, and the G120 residue on its C-terminus becomes exposed^28^. This form is designated as MAP1LC3B-I and is present in the cytosol. Then, the G120 residue is conjugated to the head group amine of phosphatidylethanolamine by the sequential action of ATG7 (an E1-like enzyme), ATG3 (an E2-like enzyme), and the ATG5–ATG12–ATG16L1 complex (an E3-like enzyme)^29^. This phosphatidylethanolamine-conjugated form, called MAP1LC3B-II, associates with autophagosome membranes and is enriched in autophagosomes. Therefore, MAP1LC3B is the best-known marker of autophagosome formation^24^. Other members of the ATG8 family undergo the same lipid conjugation modification^30^. It has been reported that MAP1LC3 and GABARAP subfamily proteins are involved in every aspect of autophagosome formation but have distinct roles. MAP1LC3 proteins are responsible for phagophore membrane elongation, while the GABARAP family is involved in downstream autophagosome maturation^31,32^. Since ULK1 can interact with members of both subfamilies^33,34^, it is possible that ULK1 has roles in both the early and late stages of autophagy in mammals. However, the potential roles of ULK1 in the late stages of autophagosome formation remain largely unknown.

We have previously established a model of autophagic cell death in adult hippocampal neural stem (HCN) cells following insulin withdrawal. HCN cells were derived from the hippocampus of a 2-month-old adult rat and have intact apoptotic capability^35^. Nevertheless, insulin withdrawal elicited cell death without signs of apoptosis, such as caspase-3 activation or chromosomal DNA condensation^35^. The pan-caspase inhibitor Z-VAD failed to reduce cell death in insulin-deprived HCN cells (designated as I(−) HCN cells, whereas HCN cells growing in the media containing insulin are hereafter designated as I(+) HCN cells, as in our previous reports)^36^. Furthermore, I(−) HCN cells had a markedly increased number of autophagic vacuoles and increased autophagy flux, and *Atg7* knockdown substantially attenuated cell death^35,37–39^. Due to the intimate involvement of glycogen synthase kinase 3 beta (GSK3B) in neuronal cell death, neurodegeneration, metabolism, and insulin signaling^40^, we hypothesized that GSK3B may positively regulate autophagy induction and subsequent cell death in HCN cells. As expected, both pharmacological and genetic inactivation of GSK3B significantly reduced autophagic cell death, while activation of GSK3B substantially increased autophagy flux and cell death without inducing apoptosis in I(−) HCN cells^41^.

GSK3B has been reported to regulate the induction of autophagy through ULK1 following growth factor deprivation^42^. However, the molecular basis of GSK3B-mediated ULK1 regulation in autophagy remains to be further elucidated. Here, we identified a novel GSK3B-mediated mechanism for ULK1 activation using HCN cells as a model for insulin withdrawal-induced autophagy. We found that GSK3B interacted with and directly phosphorylated ULK1 at residues S405 and S415. To the best of our knowledge, this is the first report of GSK3B-mediated ULK1 phosphorylation. Phosphorylation of these sites was important for the interaction of ULK1 with GABARAPL1 and MAP1LC3B. Furthermore, a marked increase in the phosphorylation of ULK1 on these residues was observed in human pancreatic cancer cell lines, suggesting the potential importance of this novel ULK1 phosphorylation for tumorigenesis in the pancreas.

## Materials and methods

### Cell culture

Adult rat HCN cells were isolated from 2-month-old Sprague-Dawley rats and cultured as reported previously^36^. Briefly, HCN cells were cultured in Dulbecco’s modified Eagle’s medium (DMEM)/F-12 (Gibco, 12400-024) supplemented with N2 components and basic fibroblast growth factor (20 ng/ml, Peprotech, 100-18B-500). Insulin was omitted to prepare an insulin-deficient medium.

HPNE cells (hTERT-immortalized acinar-to-ductal intermediary cells isolated from the adult pancreas) and BxPC3, MIA PaCa-2, and CFPAC cells (human pancreatic adenocarcinoma cell lines from the American Type Culture Collection (ATCC)) were kindly provided by Yun-Hee Kim (National Cancer Center, Korea). The HPNE cells were cultured in 75% DMEM (HyClone, SH30022.01) with 25% Medium M3 Base (Incell, M300F-100) containing 10% fetal bovine serum (FBS) and 1% penicillin/streptomycin (Gibco, 15140122). The BxPC3 cells were cultured in RPMI 1640 (HyClone, SH30027.01) containing 10% FBS and 1% penicillin/streptomycin. The MIA PaCa-2 cells were cultured in DMEM containing 10% FBS and 1% penicillin/streptomycin. The CFPAC cells were maintained in Iscove’s modified Dulbecco’s medium (HyClone SH30228.01) containing 10% FBS and 1% penicillin/streptomycin. All cells were maintained in a humidified atmosphere of 5% CO_2_ at 37 °C.

### Reagents and antibodies

Antibodies against the following proteins and epitopes were used: ACT**B**/β-actin (sc47778, HRP-conjugated), HA-tag (sc-7392), and ULK2 (sc10909) from Santa Cruz Biotechnology; phosphorylated ATG13 (S318; 600-401-C) from Rockland; BECN1 (3738S), GABARAP (13733S), GSK3B (9315S), ULK1 (8054S), and phosphorylated ULK1 (S757; 14202S) from Cell Signaling Technology; phosphorylated BECN1 (S14; 254515) from Abbiotec; ATG13 (18258-1-A), GABARAPL1 (11010-1-AP), and GABARAPL2 (18724-1-AP) from Proteintech; MAP1LC3B (NB100-2220) from Novus Biologicals; and KAT5 (bs-13686R) from Bioss. The following reagents were used: BafA_1_ (BML-CM110-0100) from Enzo Life Sciences and BIO (B1686) from Santa Cruz Biotechnology.

### Cell death assay

HCN cells were seeded in 96-well plates at a density of 5 × 10^4^ cells/cm^2^. Cell death was measured by using Hoechst 33342 (Invitrogen, H3570) and propidium iodide (PI; Sigma-Aldrich, P4170). After adding the diluted Hoechst and PI solutions to the wells and incubating the cells in the dark for 15 min, the cells were imaged under a fluorescence microscope (Axiovert 40 CFL; Carl Zeiss), and the dead cells in the collected images were counted using NIH ImageJ software. The percentage of cell death was calculated as follows:

Cell death (%) = (number of PI-positive cells [red]/total cell number [blue]) ×100

For HPNE and pancreatic cancer cells, cell death was determined by ANXA5/annexin A5 and PI co-staining at the indicated time points using an LSR-Fortessa FACS analyzer (BD Biosciences).

### DNA plasmids and transfection

GFP-ZFYVE1 (38269, deposited by Noboru Mizushima), GFP-MAP1LC3B (21073, deposited by Tamotsu Yoshimori), and ptfLC3 (encoding mRFP-GFP-MAP1LC3B, 21072, deposited by Tamotsu Yoshimori) were purchased from Addgene. GFP-GABARAPL1 (EX-Mm07891-M03) was purchased from Genecopoeia. mCherry-ULK1 was generated by cloning ULK1 WT into the pmCherry-1 vector after the deletion of ZFYVE1 from mCherry-ZFYVE1 (Addgene, 86746, deposited by Do-Hyoung Kim). To generate mCherry-ULK1 mutants, mCherry-ULK1 WT was mutated by using an EZ Mutation Site-Directed DNA Mutagenesis Kit (Enzynomics, EZ004S). ON-TARGETplus SMARTpool Rat siRNAs (a mixture of 4 siRNAs provided as a single reagent) specific to GSK3B (L 080108 02 0005), KAT5 (L 084956 02 0005), Ulk1 (L 081408 02 0005), and Ulk2 (L 083669 02 0005) were purchased from Dharmacon. The GSK3B-CA and mouse Ulk1 WT constructs were kind gifts from Yun-Il Lee (DGIST, Republic of Korea) and Joungmok Kim (Kyung Hee University, Republic of Korea), respectively.

HCN cells were transfected with Lipofectamine 2000 (Invitrogen, 11668019) according to the manufacturer’s instructions.

When we transfected ULK1^S405A^ and ULK1^S415A^, we doubled the amount of DNA to achieve similar expression levels.

### Generation of CRISPR-Cas9-mediated knockout cells

The guide RNA (gRNA) sequence for rat *Ulk1* (NCBI Gene ID: 360827), 5′-CGCGCGGCGGCGTCGAGACCGT-3′, was designed and purchased from ToolGen (Republic of Korea). To generate knockout cell lines, HCN cells were transfected with Cas9 (1 μg) and gRNA-encoding plasmid (3 μg) using Lipofectamine 2000 reagent according to the manufacturer’s guidelines. After incubation for 48 h, *Ulk1* KO cells were selected by incubation with hygromycin (300 μg/ml; Enzo Life Science, ALX-380-306-G001) for 24 h.

### Immunocytochemistry

HCN cells were fixed with 4% paraformaldehyde (Sigma-Aldrich, P6148) for 10 min and permeabilized with 0.1% Triton X-100 (Sigma, X-100) in phosphate-buffered saline (PBS). The cells were mounted in a mounting medium (Dako, S3023), and the nuclei were counterstained with Hoechst 33342. Microscopic images were obtained using an LSM780 confocal microscope (Carl Zeiss) and were analyzed using ZEN software (Carl Zeiss). The GFP-MAP1LC3B, GFP-GABARAPL1, and mCherry-ULK1 puncta were visualized with an LSM780 confocal fluorescence microscope (Carl Zeiss) and quantified by the “Analyze particles” function of ImageJ software.

### PLA assay

The proximity ligation assay was conducted using a PLA kit according to the manufacturer’s protocol. After fixation with PFA, cells were incubated with mouse anti-HA (Santa Cruz, sc-7392) and rabbit anti-GSK3B (Cell Signaling, 9315S) antibodies. Following primary antibody incubation, a pair of PLA probes (Sigma, DUO92002 and DUO92004) were added, and probe ligation, signal amplification (Sigma, DUO92007), and mounting (Sigma, DUO82040) were performed according to the manufacturer’s instructions. Representative images of the PLA signal were obtained using a confocal microscope, and the number of PLA puncta per cell was counted.

### Western blotting analysis

Cells were harvested in cold PBS at the time points indicated in the figures and lysed in radioimmunoprecipitation assay buffer (ThermoFisher Scientific, 89900) containing a protease and phosphatase inhibitor cocktail (ThermoFisher Scientific, 78441). Following centrifugation (12,000 × *g*, 15 min), protein concentrations were determined with the BCA Protein Assay Reagent (ThermoFisher Scientific, 23224). Proteins were separated by electrophoresis and electrotransferred to polyvinylidene fluoride membranes (Millipore, 1PVH00010) in a semi-dry electrophoretic transfer cell (Bio-Rad). The membranes were blocked for 1 h at room temperature in a blocking buffer consisting of 5% skim milk (Sigma-Aldrich, 70166) and 0.1% Tween 20 in Tris-buffered saline (TBST) and were then incubated at 4 °C with primary antibodies diluted 1:1000 in TBST containing 5% bovine serum albumin and 0.01% sodium azide. The membranes were washed with TBST three times for 10 min each, followed by 1 h incubation with peroxidase-conjugated secondary antibodies diluted in blocking solution. After washing, the membranes were analyzed using either Pierce ECL Western Blotting Substrate (ThermoFisher Scientific, 32106) or WesternBright ECL (Advansta, K-12045-D50).

### Generation of recombinant ULK1 fragments and in vitro kinase assay

Mouse Ulk1 (amino acid residues 279–430) WT and the S405A and S415A Ulk1 mutant constructs were cloned in pGEX-5X-3, transformed into competent *E. coli* BL21 cells, and expressed as GST-tagged proteins. Expression was induced with 1 mM isopropyl β-d-thiogalactopyranoside (Duchefa, I1401) overnight at 16 °C. Then, the cells were lysed in GST binding buffer (20 mM Tris-HCl, pH 7.5, 100 mM NaCl, 0.1% Nonidet P-40, 1 mM EDTA, and 1 mM phenylmethylsulfonyl fluoride) with 10 mg/ml lysozyme (Sigma-Aldrich, L6876) for 30 min at room temperature and centrifuged. The supernatants containing the recombinant proteins were incubated with Glutathione Sepharose 4B beads (GE Healthcare, 52-2303-00AK) overnight at 4 °C, washed with binding buffer without lysozyme, and eluted with GST elution buffer (20 mM Tris-HCl, pH 7.5, 500 mM NaCl, 0.5% Nonidet P-40, 1 mM EDTA, 1 mM phenylmethylsulfonyl fluoride, and 20 mM glutathione).

HEK293 cells were transfected with HA-GSK3B-CA, and cell lysates were subjected to immunoprecipitation with mouse anti-HA antibody for 6 h followed by overnight incubation with Protein A beads (ThermoFisher Scientific, 20333) at 4 °C. The next day, beads with bound HA-tagged GSK3B were precipitated by centrifugation, washed with PBS, and suspended in lysis buffer (20 mM Tris-HCl, pH 7.5, 50 mM NaCl, 1 mM EDTA, 250 mM sucrose, and 1% Triton X-100) containing a phosphatase inhibitor cocktail (Thermo Scientific, 78427). Kinase reaction buffer was composed of 20 mM Tris-HCl, 10 mM MgCl_2_, 5 mM dithiothreitol, and 0.4 mM NaF at pH 7.2. The kinase reaction was started by adding 200 µM ATP and recombinant mouse GST-ULK1 (279–430) protein for 40 min at 37 °C. The reaction was stopped by adding SDS sample loading buffer and boiling. The samples were subjected to western blot analysis using anti-phospho-ULK1 S405 and S415 antibodies.

### Immunoprecipitation assay

Transfected HCN cells were lysed with immunoprecipitation buffer containing 20 mM Tris-HCl (pH 7.5), 50 mM NaCl, 1 mM EDTA, 250 mM sucrose, 1% Triton X-100 and a phosphatase inhibitor cocktail (Thermo Scientific, 78427). The lysates were mixed with anti-GSK3B or anti-HA-tag antibody and incubated overnight at 4 °C, and the immune complexes were immunoprecipitated with Protein G magnetic beads (Bio-Rad, 161-4023). The immunoprecipitates were washed 5 times with lysis buffer and then denatured by adding SDS sample buffer for 10 min at 100 °C.

### Generation of phospho-specific antibodies

Anti-phospho-ULK1 S405 and anti-phospho-ULK1S415 rabbit polyclonal antibodies were raised against synthetic phosphorylated peptides encompassing S405 (ESHGRTPSP(p)SPTC) and S415 (CSSSPSP(p)SGRPGP) of mouse ULK1. The phosphopeptides were conjugated with keyhole limpet hemocyanin and injected into rabbits over an 8-week period. Polyclonal antibodies were purified by peptide-conjugated affinity chromatography.

### Statistical analysis

All values are presented as the mean ± standard error of the mean (SEM), with the results averaged over at least three independent experiments. Statistical significance was determined using the paired *t*-test for two-group experiments. For comparison of three or more groups, a one-way analysis of variance (ANOVA) followed by Tukey’s multiple comparison test was used. Differences were considered statistically significant when *P* < 0.05.

## Results

### ULK1 regulates autophagy induction in HCN cells

**Fig. 1:**
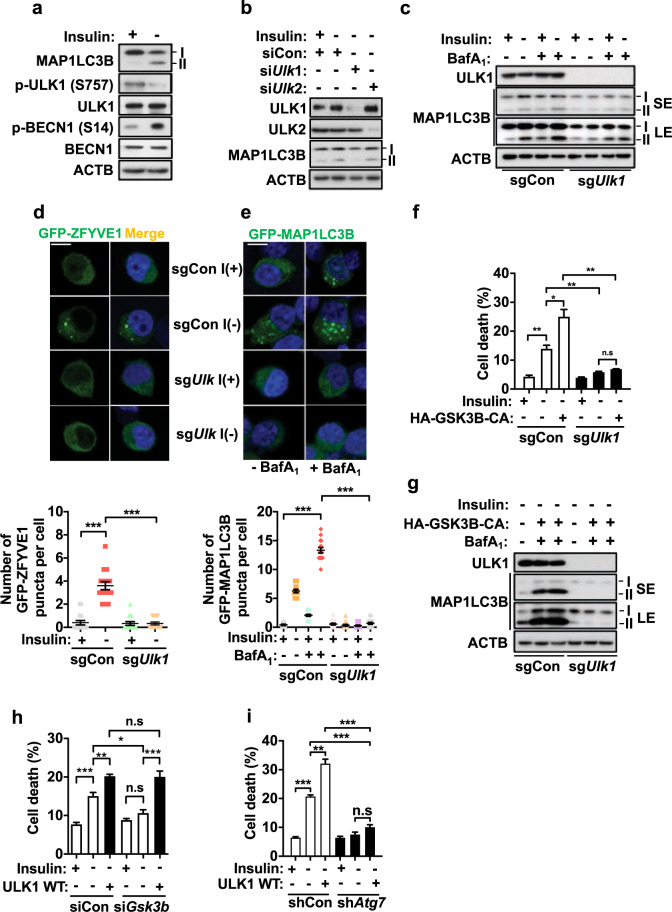
ULK1 regulates autophagy induction in HCN cells. **a** Western blotting analysis of ULK1 activation in HCN cells after insulin withdrawal for 6 h. The total and phosphorylated bands were detected in two different gels with the same lysates. **b** Western blotting analysis of the effects of ULK1 and ULK2 knockdown on MAP1LC3B lipidation in HCN cells after insulin withdrawal for 6 h. **c** Western blotting analysis of autophagy flux in *Ulk1* KO (sg*Ulk1*) and control (sgCon) HCN cells after insulin withdrawal for 6 h. Bafilomycin A_1_ (BafA_1_, 20 nM) was added 2 h before cell harvest. **d**, **e** Representative images of **d** GFP-ZFYE1 (*n* = 30) and **e** GFP-MAP1LC3B (*n* = 30) puncta in sg*Ulk1* HCN cells. Scale bar, 10 μm. The nuclei were counterstained with Hoechst 33342 (blue) after insulin withdrawal for 6 h. **f** Measurement of cell death in HCN cells transfected with HA-GSK3B-CA after insulin withdrawal for 24 h (*n* = 3). **g** Effect of GSK3B-CA overexpression on autophagy flux in sg*Ulk1* cells. Autophagy flux was analyzed by western blotting after insulin withdrawal for 6 h. **h**, **i** Measurement of cell death in **h** si*Gsk3b* (*n* = 5) and **i** sh*Atg7* (*n* = 3) HCN cells after ULK1 WT overexpression. Cell death was measured after insulin withdrawal for 24 h. SE short exposure; LE long exposure. *P < 0.05, **P < 0.01, and ***P < 0.001. ns not significant.

We previously demonstrated that GSK3B is required for autophagy activation and induces autophagy flux in insulin-deprived HCN cells without a change in its protein level^41^. To determine whether GSK3B also regulates autolysosome formation, we performed an mRFP-GFP-LC3 puncta assay in GSK3B KD cells. As expected, siGSK3B significantly reduced both the total number of LC3B puncta and the ratio of red puncta in insulin-deprived HCN cells (Supplementary Fig. S1a, b). To identify the downstream targets of GSK3B, we tested whether GSK3B induces autophagy through ULK1 since GSK3B was reported to activate ULK1 through K(lysine) acetyltransferase 5/Tat-interacting protein, 60 kDa (KAT5/TIP60)-mediated acetylation, thereby linking growth factor deprivation to autophagy induction^42^. First, we examined the effects of insulin withdrawal on ULK1 activation status in HCN cells. The MTORC1-mediated inhibitory phosphorylation of ULK1 on S757^16^ was substantially decreased, while the phosphorylation of Beclin 1 (BECN1), a ULK1 substrate^43^, at S14 was increased, suggesting that ULK1 was activated in I(−) HCN cells (Fig. 1a). In contrast, siRNA-mediated knockdown of ULK1 efficiently reduced autophagy flux in I(−) HCN cells (Fig. 1b). To distinguish the functional significance of ULK1 in autophagy induction from that of its closest family member ULK2, we also knocked down ULK2. Unlike ULK1 knockdown, ULK2 knockdown did not decrease MAP1LC3B lipidation in I(−) HCN cells (Fig. 1b). These data suggest that ULK1 but not ULK2 is essential for autophagy induction in HCN cells following insulin withdrawal. As a complementary approach, we also ablated the *Ulk1* gene by using the CRISPR-Cas9 method and generated *Ulk1* knockout (KO) HCN cells (designated as sg*Ulk1* cells). Consistent with the results of siRNA-mediated knockdown, deletion of *Ulk1* blocked autophagy induction (Fig. 1c). Examination of GFP-zinc finger FYVE-type containing 1/double FYVE-containing protein 1 (GFP-ZFYVE1/DFCP1) as an indicator of phagophore formation^44^ and GFP-MAP1LC3B as an indicator of autophagosome formation (Fig. 1d, e) confirmed efficient blockade of autophagy by *Ulk1* KO. We previously demonstrated that cell death rate in I(−) HCN cells correlated with the level of autophagy flux^45^. Therefore, the genetic ablation of *Ulk1* also diminished insulin withdrawal-induced cell death (Fig. 1f). Next, we examined whether *Ulk1* KO can attenuate autophagy induction in HCN cells via ectopic overexpression of a constitutively active (CA) mutant form of GSK3B. This mutant was generated by site-directed mutagenesis of S9 to A^41^. The S9 residue is a key regulatory site that undergoes inhibitory phosphorylation, and substitution with alanine prevents enzyme inactivation^46^. In line with our previous report^41^, overexpression of GSK3B-CA substantially elevated both autophagy flux and cell death in I(−) HCN cells (Fig. 1f, g). However, notably, *Ulk1* KO efficiently blocked these effects of GSK3B-CA (Fig. 1f, g). These data suggest that ULK1 mediates autophagy signaling downstream of GSK3B. To further demonstrate that ULK1 mediates autophagy signaling downstream of GSK3B, we examined whether knockdown of GSK3B could attenuate cell death following ULK1 overexpression. In contrast to the efficient blockade of GSK3B-induced cell death by *Ulk1* KO, GSK3B knockdown failed to reduce ULK1-induced cell death (Fig. 1h). These data suggest that ULK1 is downstream of GSK3B and can bypass GSK3B to mediate autophagy signaling.

ULK1 was implicated in cell death through autophagy-independent mechanisms under certain conditions^47^. To test whether ULK1-mediated cell death occurs through autophagy in insulin-deprived HCN cells, we expressed WT ULK1 in sh*Atg7* HCN cells. We previously demonstrated that silencing of *Atg7* blocked insulin withdrawal-induced death of HCN cells^35^. Consistent with our previous results, Atg7 knockdown efficiently attenuated cell death in insulin-deprived HCN cells, even after ULK1 overexpression (Fig. 1i). These data confirm that ULK1 mediates autophagic cell death in HCN cells following insulin withdrawal.

### GSK3B interacts with ULK1

**Fig. 2:**
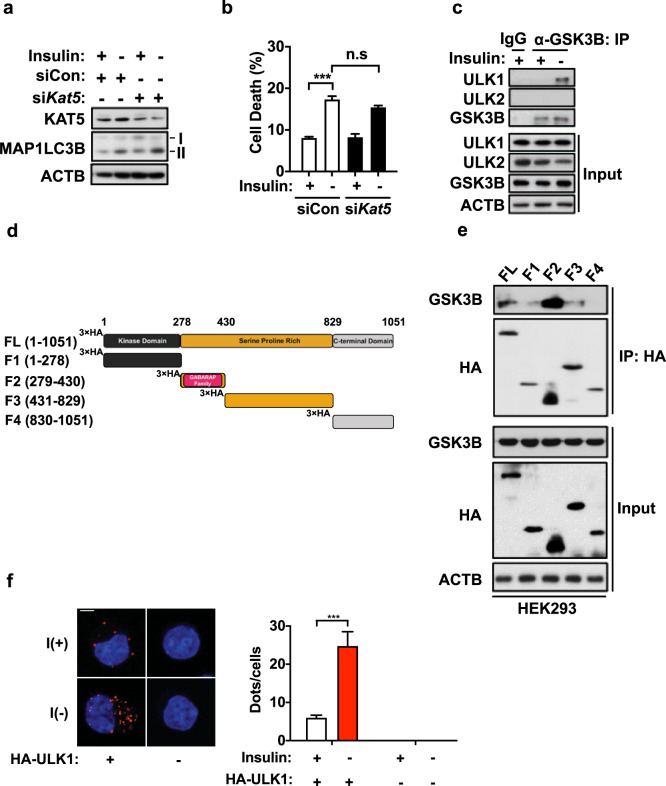
ULK1 interacts with GSK3B. **a** Western blotting analysis of the effect of KAT5 knockdown on MAP1LC3B lipidation after insulin withdrawal for 6 h. **b** Cell death rate following insulin withdrawal for 24 h in HCN cells transfected with control (siCon) or KAT5-targeting (siKAT5) siRNA (*n* = 3). **c** The interaction of endogenous ULK1 or ULK2 with GSK3B was analyzed by immunoprecipitation with anti-GSK3B antibody after insulin withdrawal for 6 h. **d** Schematic representation of the ULK1 domain structure and fragment constructs. **e** Analysis of the ULK1 regions responsible for GSK3B interaction. The indicated HA-ULK1 truncation mutants were expressed in HEK293 cells and precipitated with an anti-HA antibody, and coimmunoprecipitation with endogenous GSK3B was determined by western blotting. The blots shown are representative of at least three experiments with similar results. **f** HCN cells were fixed and analyzed by the proximity ligation assay with primary antibodies against GSK3B and the HA-tag. The cells were imaged by confocal microscopy. Scale bar, 10 μm. The graph represents the number of dots per cell following insulin withdrawal (*n* = 9 cells). ***P < 0.001. ns not significant.

To check whether KAT5 is required for autophagy induction in I(−) HCN cells, we silenced KAT5 using small interfering RNA (siRNA), but we found that KAT5 knockdown did not affect insulin withdrawal-triggered MAP1LC3B lipidation or cell death (Fig. 2a, b). After excluding the possibility of KAT5-mediated indirect ULK1 activation, we next investigated whether GSK3B directly interacts with ULK1 in HCN cells. Endogenous GSK3B was coimmunoprecipitated with ULK1 but not with ULK2, suggesting an interaction between these kinases (Fig. 2c). To identify the region of ULK1 required for interaction with GSK3B, we generated a series of ULK1 fragment mutants: F1 (N-terminal kinase domain, 1–278), F2 (GABARAP-binding region within the Serine/Proline-Rich Domain, 279–430), F3 (the rest of the Serine/Proline-Rich domain, 431–829), and F4 (C-terminal domain, 830–1051) (Fig. 2d). The GABARAP-binding region was previously identified by the Okazaki group through yeast two-hybrid screening and glutathione S-transferase (GST) pull-down assays^33^. Transient expression of the ULK1 deletion mutants in HEK293 cells and subsequent immunoprecipitation and immunoblotting analyses for endogenous GSK3B showed that only the F2 mutant retained a strong interaction with GSK3B (Fig. 2e). In addition, we detected the colocalization of GSK3B and ULK1 by conducting a proximity ligation assay (PLA), which allows quantitative visualization of protein-protein interactions. As expected, endogenous GSK3B was strongly colocalized with ULK1 after insulin withdrawal in HCN cells (Fig. 2f). Without ULK1 transfection, no PLA signal was detected. Taken together, these findings suggest that GSK3B interacts with ULK1 through the GABARAP-binding region of ULK1.

### Phosphorylation of S405 and S415 is important for ULK1 activation and autophagosome formation in response to insulin withdrawal

**Fig. 3:**
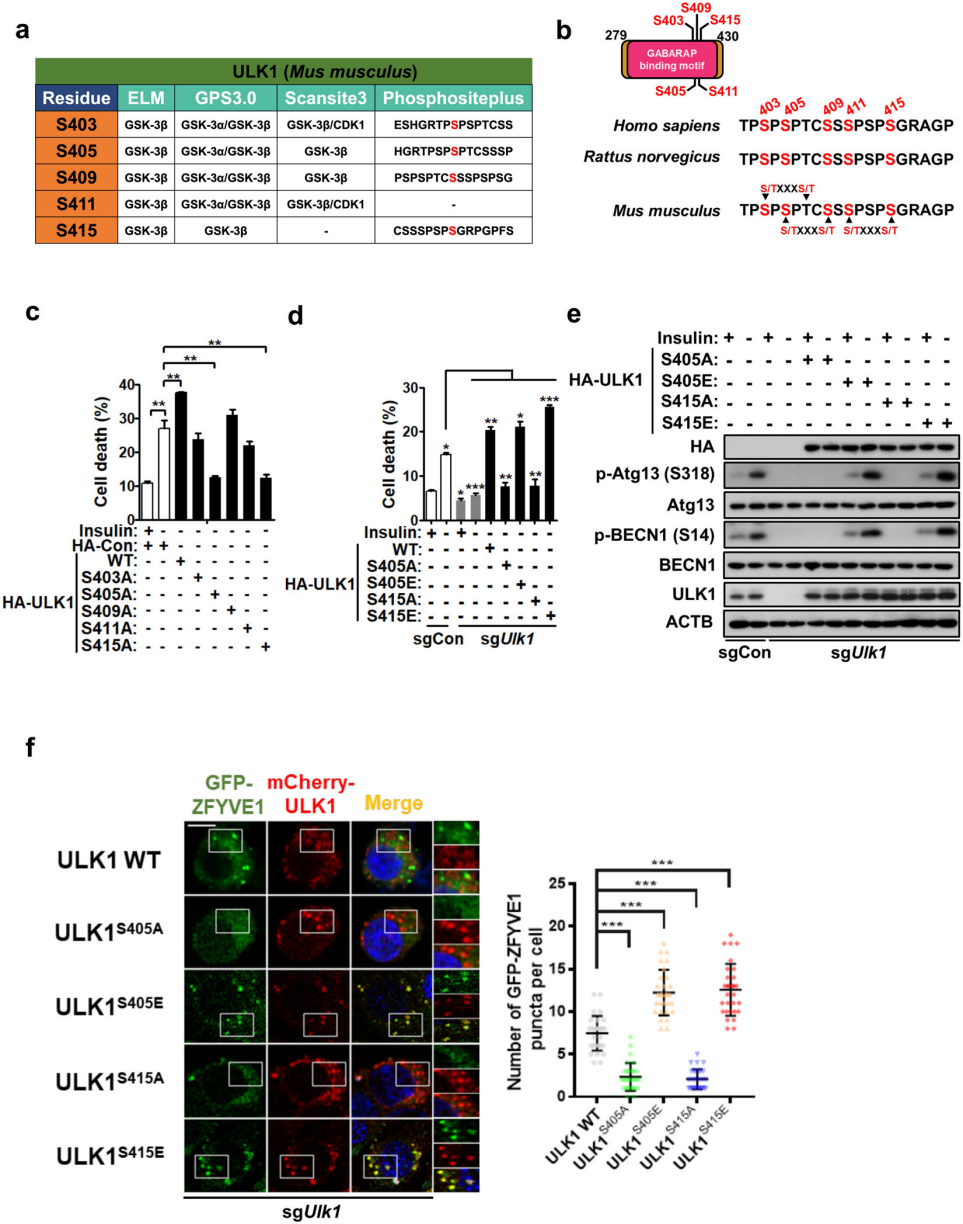
The ULK1 S405 and S415 residues are critical for ULK1 activation and autophagy induction. **a** Prediction of putative GSK3B phosphorylation sites in the Serine/Proline-Rich domain of ULK1. **b** Conservation of five candidate GSK3B phosphorylation sites in human and rodent ULK1. **c** Measurement of cell death after the expression of wild-type (WT) and phosphorylation-defective mutants of ULK1. Cell death was analyzed after insulin withdrawal for 48 h (*n* = 3). **d** Measurement of cell death after the expression of ULK1 with phosphorylation-defective or phosphorylation-mimicking mutations at the S405 and S415 residues in sg*Ulk1* HCN cells. Cell death was analyzed after insulin withdrawal for 24 h (*n* = 3). **e** Western blotting analysis of ATG13 and BECN1 phosphorylation in sg*Ulk1* HCN cells transfected with the indicated ULK1 constructs after insulin withdrawal for 6 h. The blots shown are representative of at least 3 experiments with similar results. **f** GFP-ZFYVE1 puncta assay in sg*Ulk1* HCN cells cotransfected with GFP-ZFYVE1 and the indicated mCherry-*Ulk1* constructs after insulin withdrawal for 6 h. Scale bar, 10 μm. The images were taken using an LSM780 confocal microscope (Carl Zeiss). The graph shows the quantification of puncta from three experiments (*n* = 30 cells per condition). *P < 0.05, **P < 0.01, and ***P < 0.001.

Sites phosphorylated by GSK3B can be predicted on the basis of the consensus phosphorylation sequence (S/T-X-X-X-S/T(p))^48^. To understand the functional implications of the interaction of ULK1 and GSK3B, we tried to identify the putative GSK3B phosphorylation sites in ULK1 by bioinformatics approaches using the Eukaryotic Linear Motif (ELM) website (http://www.elm.eu.org), Group-based Prediction System (GPS 3.0) algorithm (http://gps.biocuckoo.org), and Scansite3 (http://scansite3.mit.edu). The predicted residues are summarized in Supplemental Table 1. Finally, we integrated the predicted phosphorylation sites that were common among all three programs with information from the PhosphoSitePlus (PSP) database (http://www.phosphosite.org/), which provides experimentally verified posttranslational modification sites from mass spectrometry experiments^49^. Through these bioinformatics analyses, we chose five serine residues (S403, S405, S409, S411, and S415) preserved in the F2 ULK1 mutant as potential candidate residues for GSK3B-mediated phosphorylation (Fig. 3a). All these residues are highly conserved among human and rodent ULK1 sequences and are located within the GABARAP-binding region (Fig. 3b). To identify the phosphorylation site(s) critical for autophagy induction, we substituted each of the predicted serine residues with alanine using mouse *Ulk1* cDNA and examined the effects of these phosphorylation-null ULK1 mutants on cell death in I(−) HCN cells. Of the five potential phosphorylation sites, mutation of S405 and S415 effectively prevented HCN cell death for up to 48 h following insulin withdrawal (Fig. 3c). To further validate S405 and S415 as GSK3B target sites, we expressed phosphorylation-defective or phosphorylation-mimicking point-mutated forms of ULK1 in sg*Ulk1* HCN cells. ULK1 overexpression was reported to mildly inhibit starvation-induced autophagy in HEK293 cells^10^. Therefore, to avoid the confounding effects of endogenous ULK1 and achieve expression of WT and mutant ULK1 at comparable levels, we transfected *Ulk1* WT, S405, or S415 mutant constructs into sg*Ulk1* cells. The expression of *Ulk1* WT rescued cell death in insulin-deprived sg*Ulk1* cells (Fig. 3d). As expected, expression of the phosphorylation-mimicking *Ulk1* S405E or S415E mutant also increased cell death (in the case of the S415E mutant, to a level even slightly higher than that of *Ulk1* WT); however, the introduction of the *Ulk1* S405A or S415A mutant failed to decrease the cell death rate (Fig. 3d). Cells transfected with an *Ulk1* S405A/S415A double mutant behaved similarly to those transfected with the single mutants. The double mutant decreased cell death (from 25.7 ± 1.0% to 8.2 ± 1.2%; mean ± SEM, *n* = 3) after insulin withdrawal for 24 h, similar to the S405A (8.5 ± 1.4%) and S415A (8.7 ± 1.2%) mutants. Ectopic expression of the constructs was confirmed by western blotting analysis with an antibody against the hemagglutinin (HA) epitope tag (Fig. 3e). These data suggest that these two putative phosphorylation sites are indispensable for ULK1 activation and subsequent autophagy induction following insulin withdrawal in HCN cells. To test whether phosphorylation of S405 or S415 is required for ULK1 activation, we monitored the phosphorylation of BECN1 S14 and ATG13 S318 as indicators of ULK1 activity. Consistent with our hypothesis that S405 or S415 phosphorylation is required for ULK1 activation, increased phosphorylation of all these sites was detected in sg*Ulk1* cells reconstituted with S405E or S415E ULK1, but not in cells reconstituted with S405A or S415A ULK1 (Fig. 3e). To examine the role of phosphorylation of S405 or S415 in autophagy induction, the formation of ZFYVE1 puncta was assessed after transient expression of GFP-ZFYVE1 in sg*Ulk1* HCN cells. After insulin withdrawal, many GFP-ZFYVE1 puncta were formed in sg*Ulk1* HCN cells expressing ULK1 WT and both phosphomimetic mutants, whereas the S405A and S415A mutants did not rescue ZFYVE1 puncta formation (Fig. 3f). Furthermore, an increase in the number of GFP-ZFYVE1 puncta was accompanied by overlap with ULK1 puncta, confirming the dynamic association of the ULK1 phosphomimetic and WT forms with the early autophagosome structure (Fig. 3f). These results indicate that phosphorylation of S405 or S415 is required for ULK1 activation and subsequent autophagy induction in HCN cells following insulin withdrawal.

### GSK3B phosphorylates ULK1 at S405 and S415

**Fig. 4:**
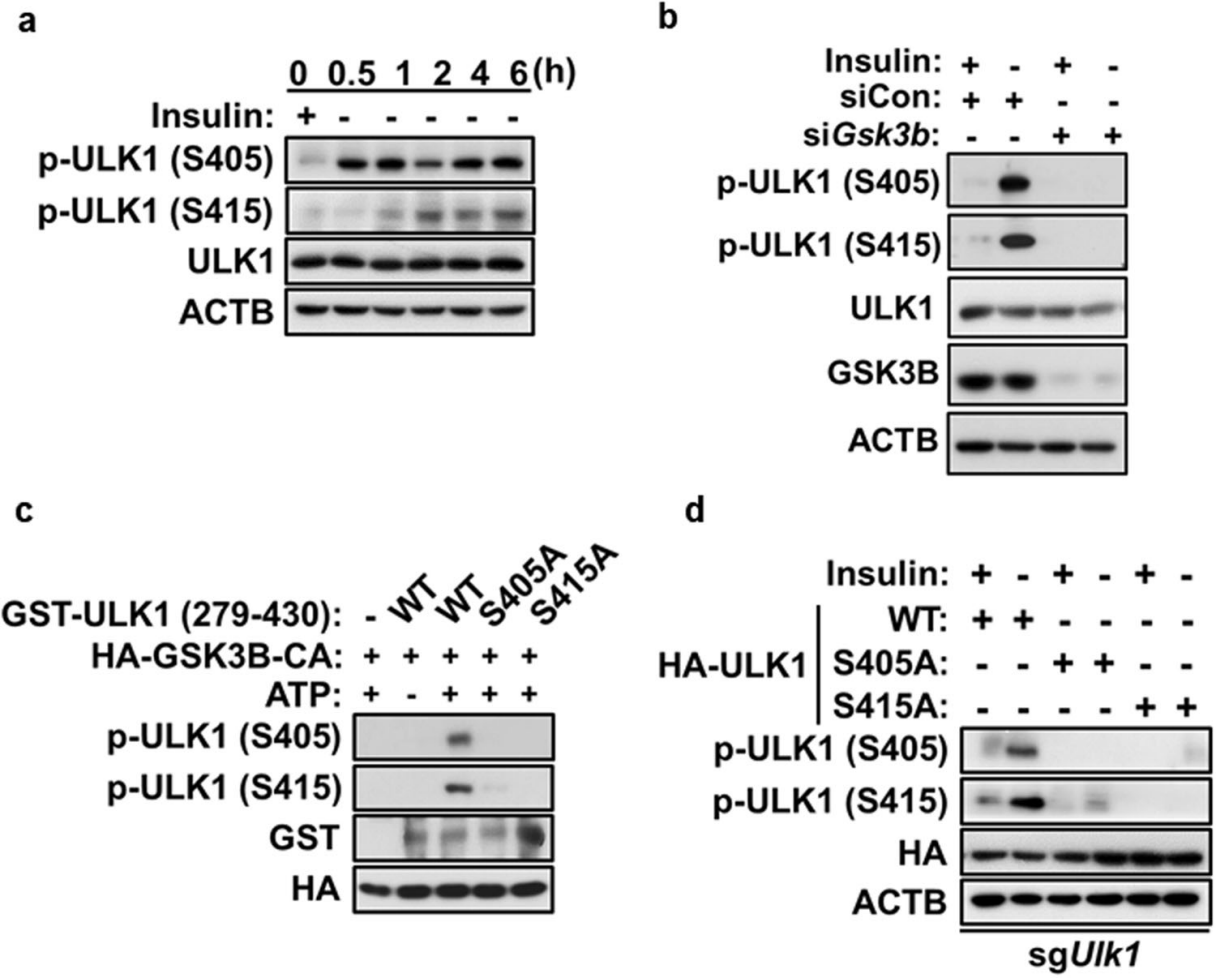
GSK3B phosphorylates ULK1 at S405 and S415. **a** Time-course analysis of ULK1 phosphorylation. The western blotting analysis was performed with S405 and S415 phospho-specific antibodies following insulin withdrawal. **b** Effect of GSK3B knockdown on ULK1 S405 and S415 phosphorylation after insulin withdrawal for 6 h. **c** In vitro kinase assay for ULK1 phosphorylation at S405 and S415. GST-tagged ULK1 fragments (amino acid residues 279–430) with mutations at S405A or S415A were used as substrates for HA-GSK3B-CA immunoprecipitation from HEK293 cells. The western blotting analysis was performed with S405 and S415 phospho-specific antibodies. **d** Effect of S405 or S415 phosphorylation on the phosphorylation of the other. The ULK1 WT, S405A, or S415A construct was expressed in sg*Ulk1* cells, and western blotting analysis using phospho-specific antibodies was performed after insulin withdrawal for 6 h. In all experiments, the blots shown are representative of at least three experiments with similar results.

To assess the phosphorylation of endogenous ULK1 on S405 and S415, we generated polyclonal antibodies specific to the phosphorylated S405 or S415 site. Western blotting and immunostaining analyses with these antibodies revealed much higher levels of S405 and S415 phosphorylation in I(−) than I(+) HCN cells (Supplementary Fig. S2a, b). Time-course analyses showed that insulin withdrawal-induced robust ULK1 phosphorylation at S405 as early as 30 min (the earliest time point we examined), and the phosphorylation level remained elevated thereafter. In contrast, phosphorylation of S415 became detectable at 1 h and then gradually increased, revealing different phosphorylation time courses between S405 and S415 following insulin withdrawal (Fig. 4a). To determine whether ULK1 phosphorylation at S405 and S415 is dependent on GSK3B, we knocked down GSK3B using siRNA and found that it abolished ULK1 phosphorylation (Fig. 4b). These data confirmed that phosphorylation of ULK1 at S405 and S415 occurred in a GSK3B-dependent manner. To demonstrate that S405 and S415 are directly phosphorylated by GSK3B rather than by other kinases triggered by GSK3B, we performed an in vitro kinase assay with the GST-tagged ULK1 F2 fragment and immunoprecipitated GSK3B-CA. We expressed the F2 fragment, which contains S405 and S415, to improve the efficiency of expression and purification, since ULK1 is a very large protein (1051 amino acid residues). We expressed HA-GSK3B-CA in HEK293 cells and immunoprecipitated it with an anti-HA antibody. The GST-ULK1 (279–430) WT, S405A, and S415A recombinant proteins were purified from *Escherichia coli* using glutathione-agarose beads and incubated with the GSK3B immune complex in the presence of ATP. S405 and S415 phosphorylation of GST-ULK1 was probed with antibodies specific to each phosphorylated residue. As expected, the GSK3B immune complex phosphorylated ULK1 WT but not the S405A or S415A mutant (Fig. 4c). Interestingly, the S415A mutation completely prevented S405 phosphorylation, while the S405A mutation led to substantial but incomplete prevention of S415 phosphorylation, which suggests that there is crosstalk between these two sites (Fig. 4d). These results identified S405 and S415 of ULK1 as novel GSK3B phosphorylation sites critical for the induction of autophagy in HCN cells following insulin withdrawal.

### Phosphorylation of S405 and S415 promotes the interaction of ULK1 with GABARAPL1 and MAP1LC3B

**Fig. 5:**
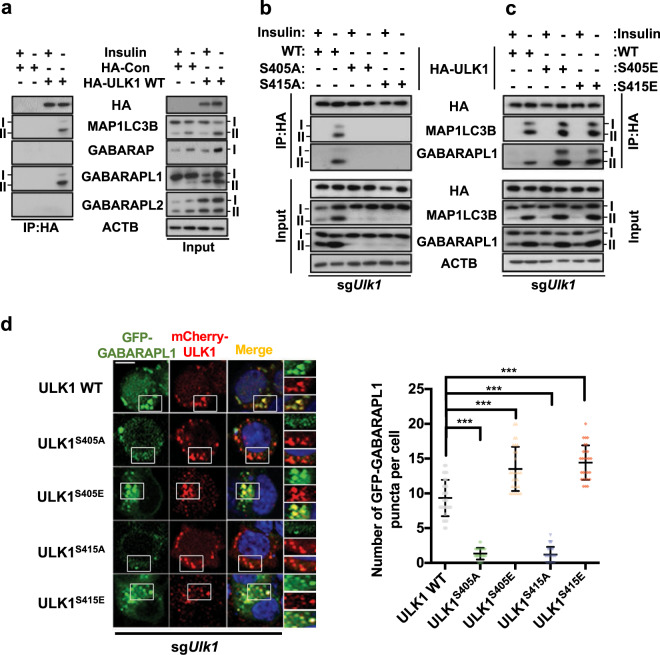
Phosphorylation of S405 and S415 promotes the interaction of ULK1 with GABARAPL1. **a** Interaction of endogenous MAP1LC3B and GABARAPL1 with HA-ULK1 WT. HCN cells were transfected with HA-ULK1 WT, and immunoprecipitation was performed with an anti-HA antibody after insulin withdrawal for 6 h. **b**, **c** Interaction of the phosphomimetic (S405E and S415E) (**c**) but not the phosphodeficient (S405A and S415A) mutant forms of ULK1 (**b**) with GABARAPL1 in insulin-deficient HCN cells. sg*Ulk1* HCN cells were transfected with the indicated HA-ULK1 constructs, and immunoprecipitation was performed with an anti-HA antibody after insulin withdrawal for 6 h. **d** GABARAPL1 puncta assay in sg*Ulk1* HCN cells cotransfected with the indicated mCherry-ULK1 constructs and GFP-GABARAPL1. The GFP-GABARAPL1 puncta were imaged after insulin withdrawal for 6 h using an LSM780 confocal microscope. Scale bar, 10 μm. The graph shows the quantification of puncta from three experiments (*n* = 30 cells per condition). In all experiments, the blots shown are representative of at least three experiments with similar results. ***P < 0.001.

Having established that GSK3B phosphorylates ULK1 within the motif predicted to interact with GABARAP family proteins^33^, we next sought to determine whether these sites are indeed important for ULK1 association with GABARAP proteins. Previous analyses of the interaction of ULK1 with ATG8 family proteins revealed a stronger interaction with the GABARAP subfamily than with the MAP1LC3B subfamily^33,50^. In contrast, we observed a strong preference for HA-tagged full-length ULK1 transiently expressed in HCN cells for GABARAPL1 and MAP1LC3B; these interactions were observed only following insulin withdrawal and not under I(+) conditions (Fig. 5a). The finding that ULK1 interacts with GABARAPL1 and MAP1LC3B only under insulin withdrawal conditions suggests that the phosphorylation of ULK1 is necessary for this interaction. To test this assumption, we expressed ULK1 with a phosphomimetic or phosphodefective mutation at S405 or S415 and performed immunoprecipitation following insulin withdrawal. Supporting the importance of phosphorylation at these residues, the S405E and S415E (Fig. 5c) but not the S405A or S415A (Fig. 5b) forms of ULK1 interacted with MAP1LC3B and GABARAPL1. Notably, ULK1 with S residues mutated to A residues at GSK3B phosphorylation sites did not facilitate the lipidation of MAP1LC3B or GABARAPL1 (Fig. 5b, c) or the formation of GABARAPL1 puncta (Fig. 5d). The interaction of phosphomimetic and WT ULK1 with GABARAPL1 was reflected in their increased colocalization, which indicated the recruitment of ULK1 to autophagosomes (Fig. 5d). These data suggest that GSK3B-mediated phosphorylation of ULK1 is required for its interaction with GABARAPL1 and MAPL1LC3B and autophagy induction in insulin-deprived HCN cells. Notably, the interaction of the phosphomimetic forms of ULK1 with GABARAPL1 and MAP1LC3B was observed under I(−) but not I(+) conditions (Fig. 5c). The lipidation of GABARAPL1 and MAP1LC3B by ULK1^S405E^ or ULK1^S415E^ was also marginal in the I(+) condition compared with the I(−) condition, suggesting that the phosphomimetic S405E and S415E mutations do not recapitulate the entire milieu of insulin withdrawal (Fig. 5c). These data suggest that phosphorylation of ULK1 at S405 and S415 is necessary, but not sufficient for autophagy induction in HCN cells following insulin withdrawal.

### ULK1 phosphorylation at S405 and S415 is enhanced in human pancreatic cancer cell lines

**Fig. 6:**
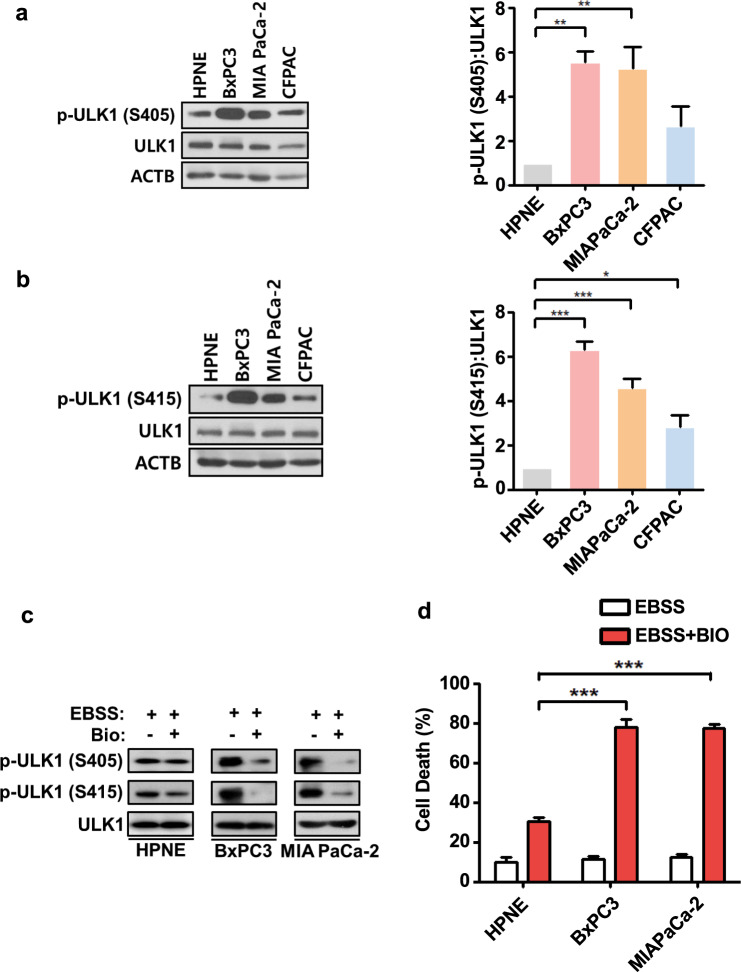
ULK1 phosphorylation is upregulated in human pancreatic cancer cell lines. **a**, **b** Western blotting analysis of a normal human pancreatic cell line (HPNE, human pancreatic nestin-expressing cells) and cancer cell lines (BxPC3, MIA PaCa-2, and CFPAC) for endogenous levels of ULK1 phosphorylation at **a** S405 and **b** S415 with either S405 or S415 phospho-specific antibodies. The graph shows the quantification of p-ULK1 levels after normalization to total ULK1 (*n* = 4). **c** Inhibition of ULK1 phosphorylation at S405 and S415 by BIO for 2 h in BxPC3 and MIA PaCa-2 cells. **d** Effect of BIO on the survival of BxPC3 and MIA PaCa-2 cells upon EBSS starvation. (*n* = 5). In all experiments, the blots shown are representative of at least three experiments with similar results. *P < 0.05, **P < 0.01, and ***P < 0.001.

To further explore the physiological implications of ULK1 phosphorylation at S405 and S415, we examined the phosphorylation levels of ULK1 in several pancreatic cancer cell lines. Pancreatic cancer is a well-known cancer type that requires active autophagy for cancer cell growth and survival^51^. High expression levels of GSK3B and robust basal autophagy flux in the pancreatic cancer cell lines BxPC3, MIA PaCa-2, and CFPAC were previously reported^52–54^. Interestingly, the levels of phosphorylation of endogenous ULK1 at both S405 and S415 in the same pancreatic cancer cell lines were significantly higher than those in normal human pancreatic ductal epithelial cells and human pancreatic nestin-expressing (HPNE) cells (Fig. 6a, b). This phosphorylation status of ULK1 is well correlated with the high expression levels of GSK3B in pancreatic cancer cell lines, compared with HPNE cells (Supplementary Fig. S2). To examine whether increased ULK1 phosphorylation in these cell lines was sensitive to GSK3 inhibition, we treated the cells with BIO, a GSK3B inhibitor that efficiently inhibits GSK3B activity in HCN cells^41^. Treatment with BIO substantially reduced ULK1 S405 and S415 phosphorylation (Fig. 6c) and rendered pancreatic cancer cells more vulnerable to starvation stress than HPNE cells (Fig. 6d). Interestingly, the total levels of ULK1 were similar in pancreatic normal and cancer cell lines. These results implicate ULK1 phosphorylation at S405 and S415 in the survival of pancreatic cancer cells.

## Discussion

**Fig. 7:**
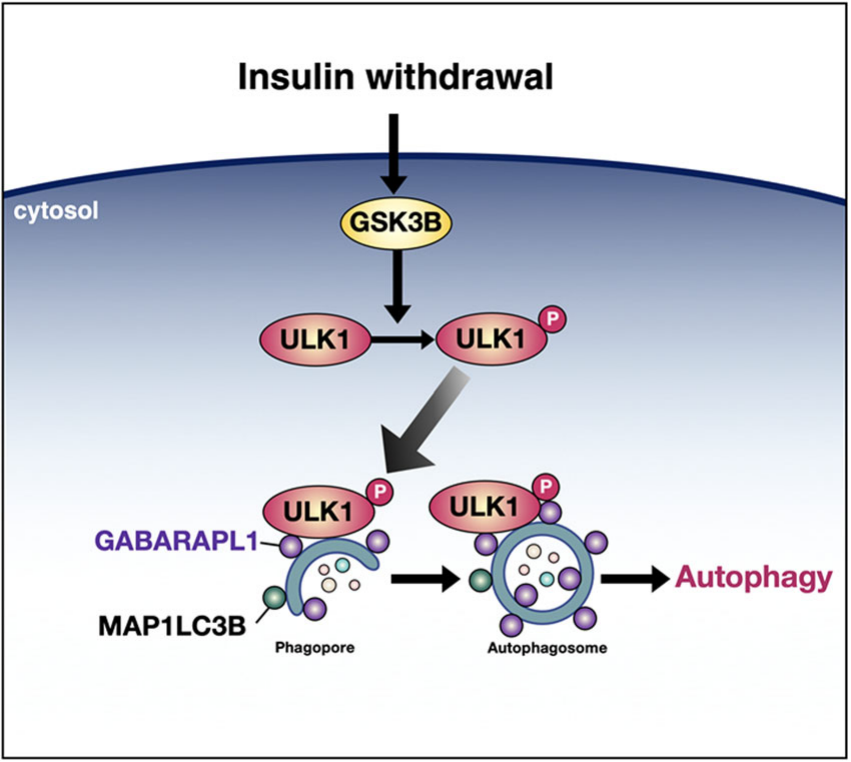
Scheme showing autophagy induction by GSK3B-mediated phosphorylation of ULK1.

Since autophagy is one of the major mechanisms by which eukaryotic cells respond and adapt to cellular stress, it is important to understand how autophagy is induced and terminated. To better understand the regulatory mechanisms of autophagy induction in response to growth factor withdrawal, we chose HCN cells as a model system since the survival of HCN cells depends on insulin and its withdrawal induces autophagic cell death. In addition, more knowledge of the autophagy machinery in HCN cells has been recently gained^55–57^. Using this autophagy induction model, we identified new regulatory mechanisms of ULK1 activation. In this report, we first showed, using biochemical and microscopic autophagy assays, that GSK3B interacts with ULK1 to induce autophagy. GSK3B-triggered autophagy induction is executed via phosphorylation of ULK1 at the novel S405 and S415 sites (Fig. 7). Phosphorylated ULK1 interacts preferentially with GABARAPL1, and accordingly, GABARAPL1 seems to have a more important role in autophagy in HCN cells than other GABARAP members.

Autophagosome formation tightly controlled by mechanisms working at multiple levels, encompassing the transcription, translation, and posttranslational modification and degradation of target proteins. Posttranslational modification of regulatory kinases is a rapid and efficient method of autophagy regulation, and one of the kinases undergoing various posttranslational modifications is ULK1. More than 30 phosphorylation sites have been suggested for ULK1^7^, and far more are listed in databases (Supplemental Table 1). The putative phosphorylation sites are distributed along all three domains of the protein; however, the kinases responsible for ULK1 phosphorylation and the functional roles of most of these sites remain to be elucidated^7^. For a further in-depth study of the importance of phosphorylation at S405 and S415, we generated phospho-specific polyclonal antibodies against phosphorylated S405 or S415. Insulin-deprived HCN cells displayed rapid phosphorylation of S405 and, subsequently, slower phosphorylation of S415. This suggests a priming role of S405 in the phosphorylation of S415. However, a point mutation of one of the two residues blocks or reduces phosphorylation of the other site, suggesting sophisticated crosstalk between these two residues that we do not yet fully understand. More analysis is needed to better define the specific role of each site and to explore possible crosstalk with other phosphorylation sites in terms of phosphorylation-mediated regulation of ULK1 activity.

Interestingly, high phosphorylation levels of ULK1 S405 and S415 were observed in several human pancreatic cancer cell lines, all of which are known to have high levels of autophagy flux. Interestingly, the levels of GSK3B are significantly upregulated in human pancreatic cancer patients, and this upregulation is closely associated with a decrease in overall survival (TCGA database; http://gepia.cancer-pku.cn). As such, inhibition of ULK1 phosphorylation at S405 and S415 in pancreatic cancer cell lines significantly decreased their survival rates under starvation conditions. Our study suggests the importance of phosphorylation at these sites as a new indicator of the autophagy level in response to nutrient and growth factor starvation and potentially in certain types of tumorigenesis.

Collectively, our data elucidate a new signaling pathway that involves GSK3B-mediated ULK1 activation and may underlie the rapid induction of autophagy in response to nutrient/growth factor stress.

## Supplementary information

Supplemental figures

Supplemental figures and table
