# Supplementary material for: GSK3B induces autophagy by phosphorylating ULK1
Source: Exp Mol Med. 2021 Mar 2;53(3):369–83. doi: 10.1038/s12276-021-00570-6 (PMC8080724; doi:10.1038/s12276-021-00570-6)
Supplement: Supplementary file 1 — Supplemental figures [file 12276_2021_570_MOESM1_ESM.pptx]

## Slide 1
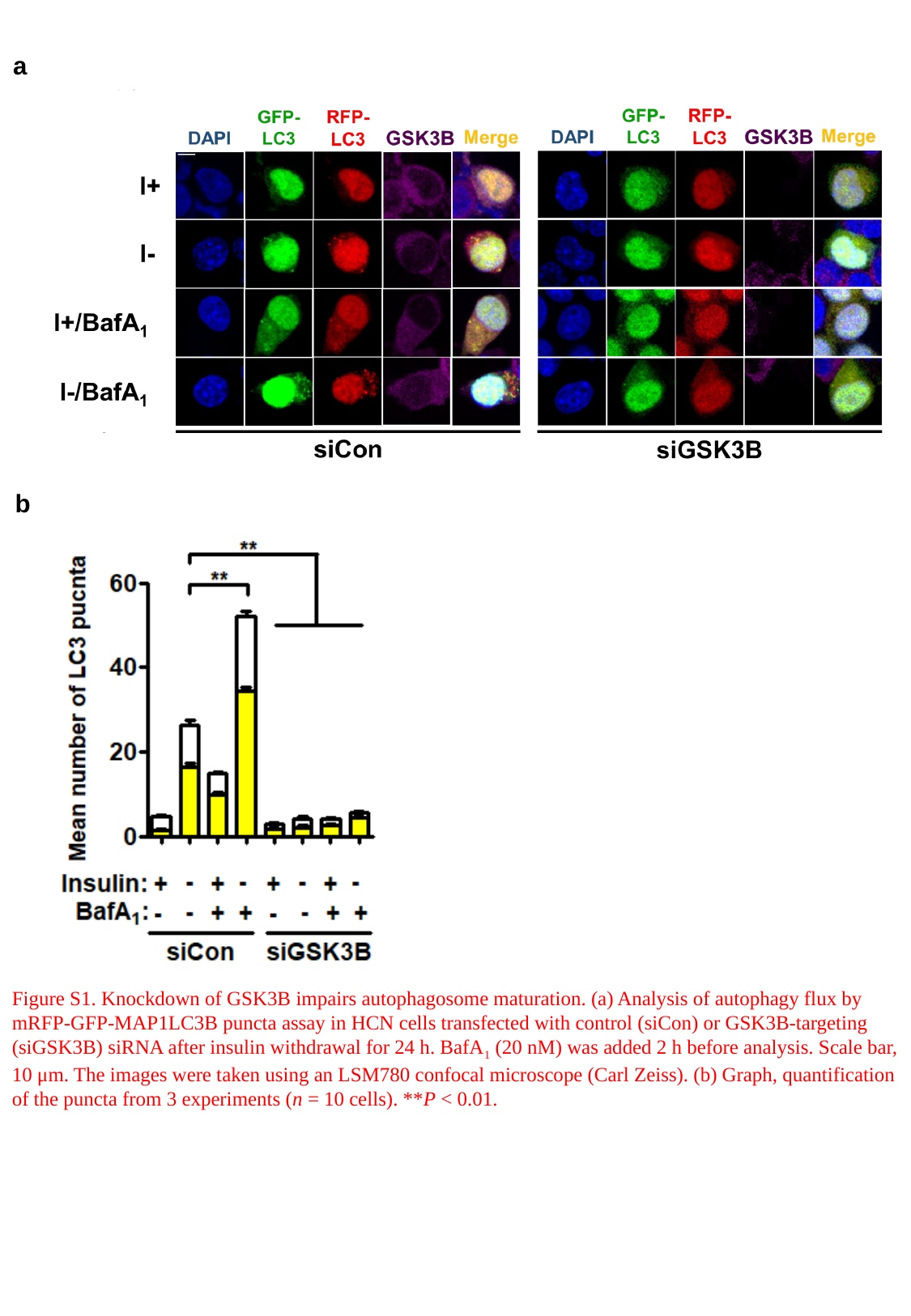

a
b
Figure S1. Knockdown of GSK3B impairs autophagosome maturation. (a) Analysis of autophagy flux by mRFP-GFP-MAP1LC3B puncta assay in HCN cells transfected with control (siCon) or GSK3B-targeting (siGSK3B) siRNA after insulin withdrawal for 24 h. BafA1 (20 nM) was added 2 h before analysis. Scale bar, 10 μm. The images were taken using an LSM780 confocal microscope (Carl Zeiss). (b) Graph, quantification of the puncta from 3 experiments (n = 10 cells). **P < 0.01.

## Slide 2
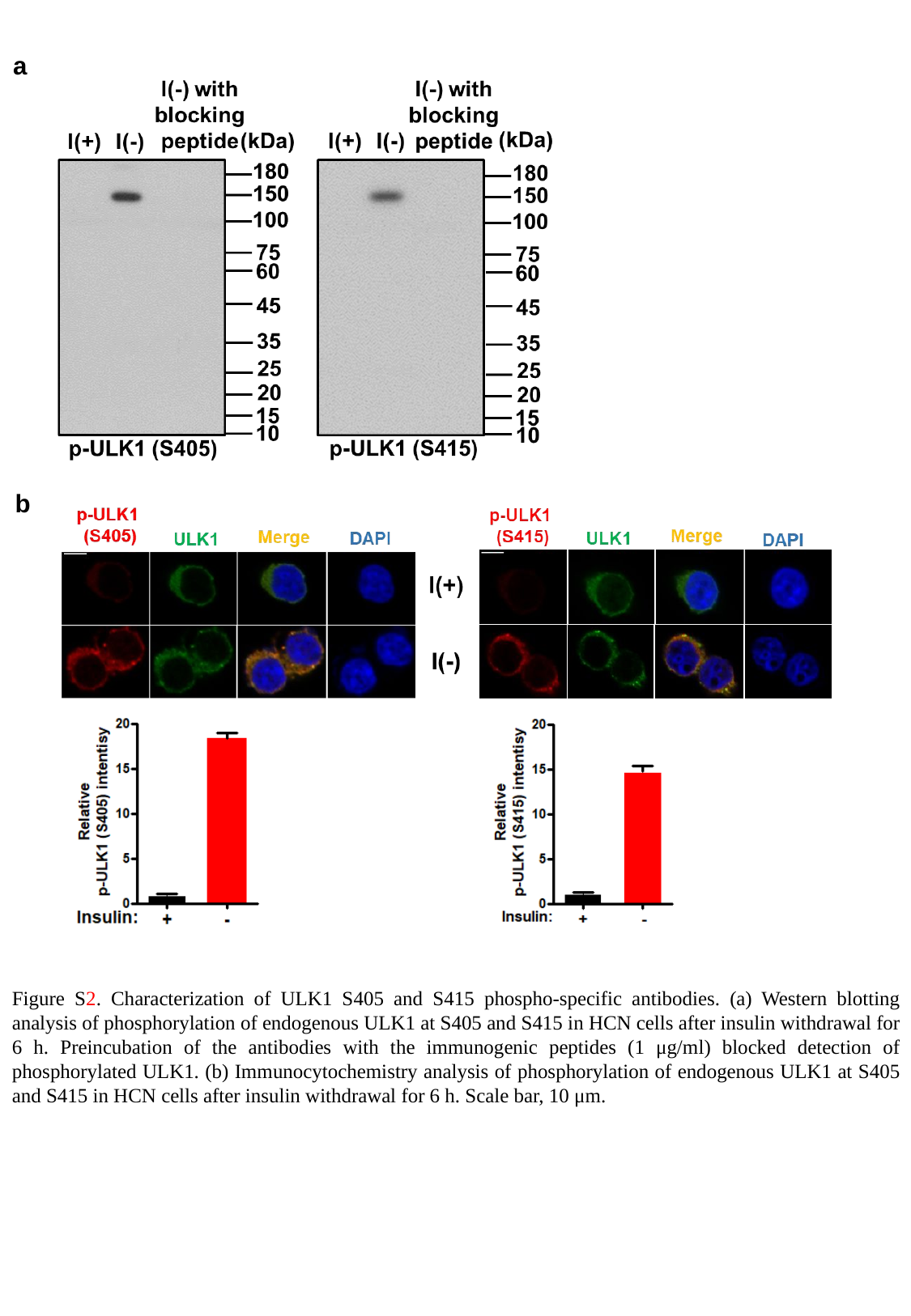

a
b
Figure S2. Characterization of ULK1 S405 and S415 phospho-specific antibodies. (a) Western blotting analysis of phosphorylation of endogenous ULK1 at S405 and S415 in HCN cells after insulin withdrawal for 6 h. Preincubation of the antibodies with the immunogenic peptides (1 μg/ml) blocked detection of phosphorylated ULK1. (b) Immunocytochemistry analysis of phosphorylation of endogenous ULK1 at S405 and S415 in HCN cells after insulin withdrawal for 6 h. Scale bar, 10 μm.
