# Supplementary material for: GSK3B induces autophagy by phosphorylating ULK1
Source: Exp Mol Med. 2021 Mar 2;53(3):369–83. doi: 10.1038/s12276-021-00570-6 (PMC8080724; doi:10.1038/s12276-021-00570-6)
Supplement: Supplementary file 2 — Supplemental figures and table [file 12276_2021_570_MOESM2_ESM.pdf]

a

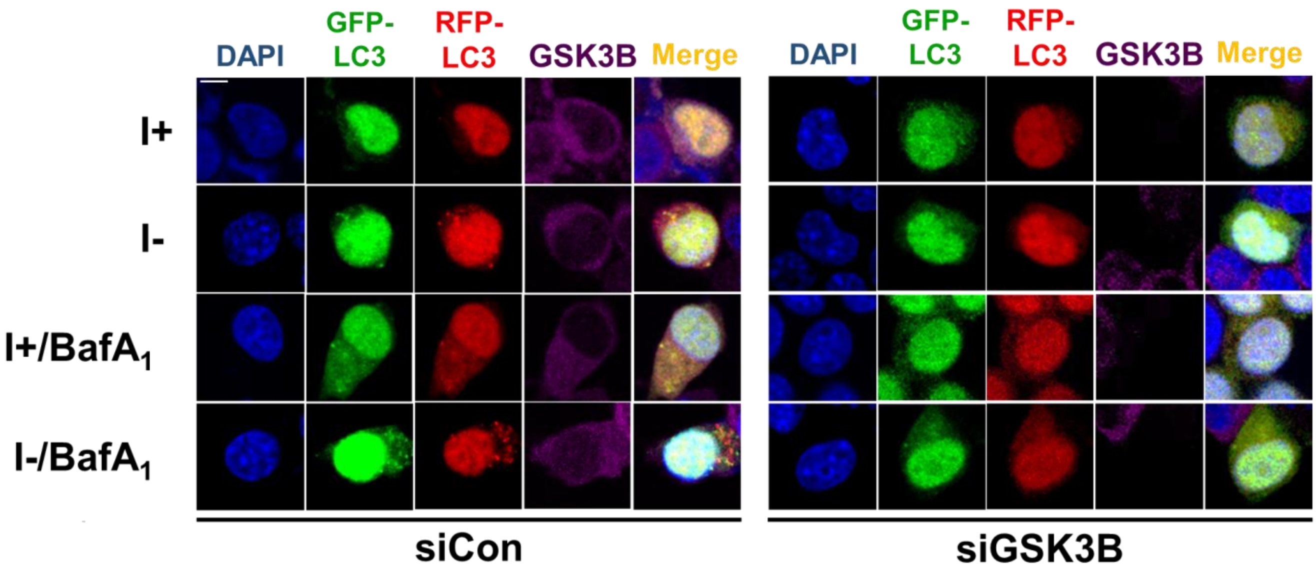

b

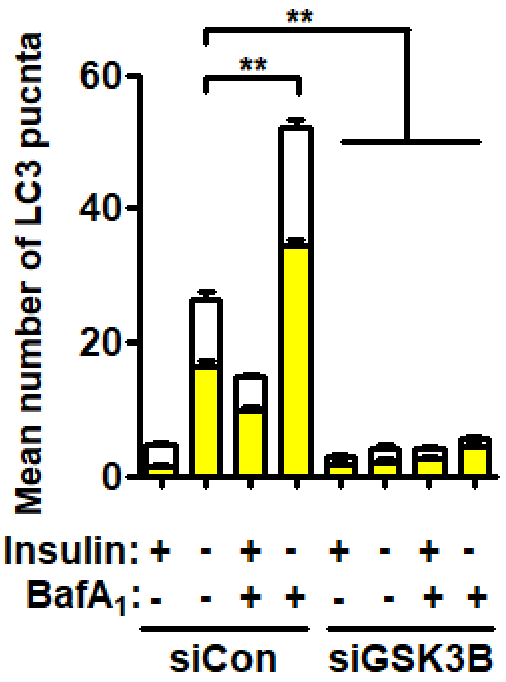

Figure S1. Knockdown of GSK3B impairs autophagosome maturation. (a) Analysis of autophagy flux by mRFP-GFP-MAP1LC3B puncta assay in HCN cells transfected with control (siCon) or GSK3B-targeting (siGSK3B) siRNA after insulin withdrawal for 24 h. $BafA_1$ (20 nM) was added 2 h before analysis. Scale bar, 10 μm. The images were taken using an LSM780 confocal microscope (Carl Zeiss). (b) Graph, quantification of the puncta from 3 experiments (n = 10 cells). **$P < 0.01$.

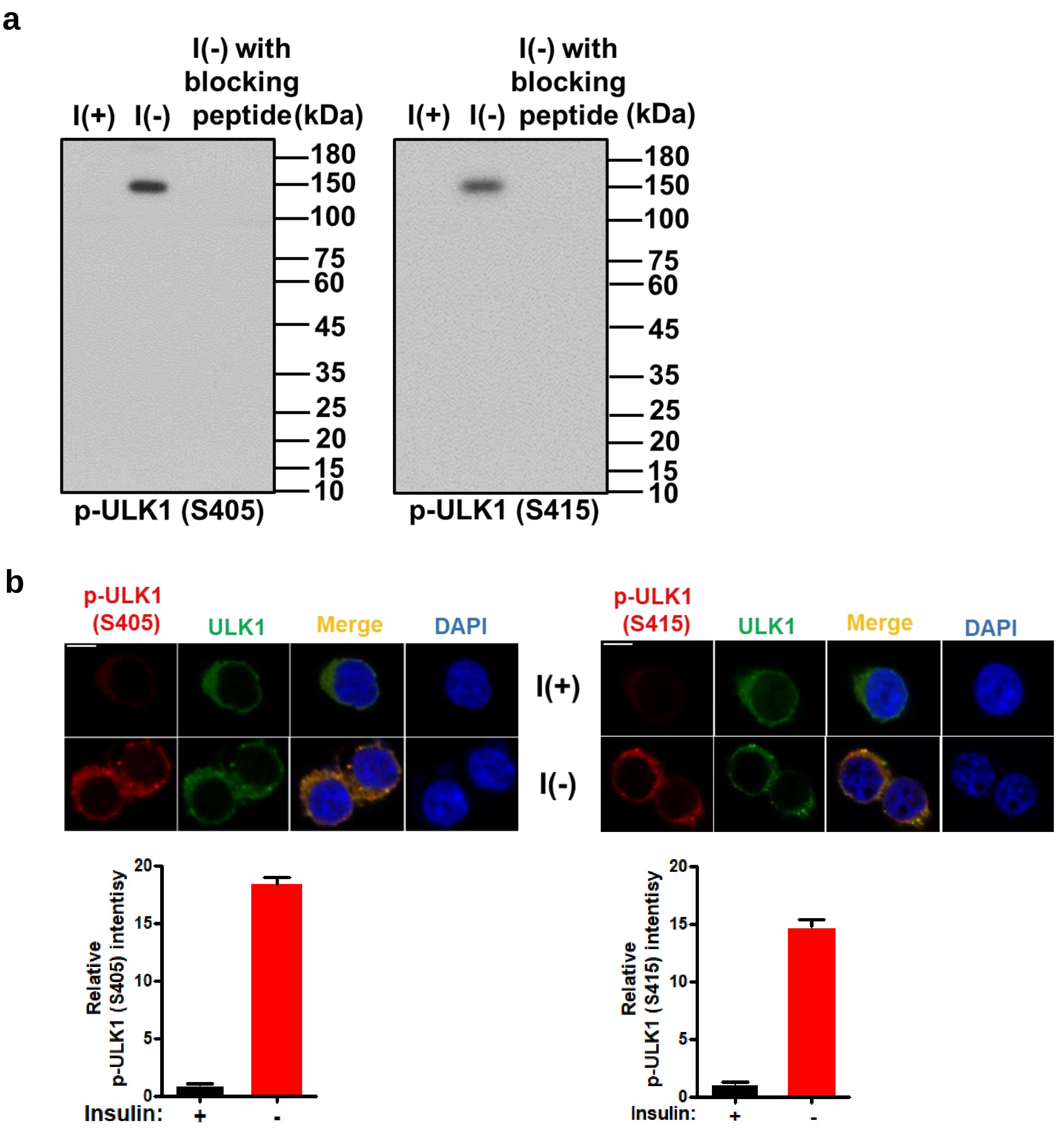

Figure S2. Characterization of ULK1 S405 and S415 phospho-specific antibodies. (a) Western blotting analysis of phosphorylation of endogenous ULK1 at S405 and S415 in HCN cells after insulin withdrawal for 6 h. Preincubation of the antibodies with the immunogenic peptides (1 μg/ml) blocked detection of phosphorylated ULK1. (b) Immunocytochemistry analysis of phosphorylation of endogenous ULK1 at S405 and S415 in HCN cells after insulin withdrawal for 6 h. Scale bar, 10 μm.

Table1. Predicted phosphorylation sites of ULK1 by GSK3B

1	ELM	GPS3.0	Scansite3	PhosphoSitePlus
2				S87
3				S111
4				S131
5				S147
6		T180		T180
7				S195
8				S224
9				S225
10				S281
11				T282
12	S294		S294	
13	S295			
14	S298		S298	
15	S300	S300		
16	S302	S302	S302	
17	S303	S303		
18	S304	S304	S304	
19	S305	S305		
20	S306			
21	S307	S307		
22	S308	S308		
23	S310	S310		
24	S314	S314		
25		S317		S317
26				S330
27	S341			S341
28	S344			
29	S347			
30	S348			
31	S351			

**32**	S381			
**33**	S385			
**34**	S387			
**35**	S388			
**36**	S392			
**37**	S397			
**38**	T401	T401	T401	
**39**	S403		S403	S403
**40**	S405	S405	S405	S405
**41**	T407	T407	T407	
**42**	S409	S409	S409	S409
**43**	S410	S410		
**44**	S411	S411	S411	
**45**	S413	S413		
**46**	S415	S415		S415
**47**				S429
**48**				
**49**				
**50**	S450			S450
**51**	T452			
**52**	T456			T456
**53**	S459			S459
**54**	S460			S460
**55**	S465			S465
**56**	S467			S467
**57**	T468			T468
**58**	T469			T469
**59**		S477		S477
**60**		S479		S479
**61**				S482
**62**				S494
**63**		T509		
**64**	S515			

**65**	S517	S517		
**66**	S521	S521		S521
**67**				S532
**68**				S537
**69**				S538
**70**	S543			S543
**71**	T546			
**72**	T547			
**73**				S555
**74**				S560
**75**				T574
**76**	T580			
**77**	S582			S582
**78**	T586	T586		
**79**				S587
**80**				S597
**81**		S604		S604
**82**		S614		S614
**83**		S622		S622
**84**				T624
**85**		T635		T635
**86**				S637
**87**				S638
**88**		T653		T653
**89**		T659		T659
**90**		S667		
**91**	S691			
**92**	S693			S693
**93**	T694			T694
**94**	S695			
**95**	T698			T698
**96**	T708			T708
**97**	S711			
**98**	S713			S713
**99**	S715			S715
**100**				T716
**101**				S718

**102**				S746
**103**				S747
**104**				S757
**105**	S760			S760
**106**	T763	T763		T763
**107**	S767			
**108**	T770			
**109**	S774			S774
**110**	S777			S777
**111**	S778			
**112**	S779			
**113**	S780			S780
**114**	S783			
**115**	T784			
**116**	S786			
**117**	S787			
**118**	S788			
**119**	T844			
**120**	S847			
**121**	T851			
**122**				S867
**123**	S913			S913
**124**	S914			
**125**	T917			
**126**				S950
**127**	S1043			S1043
**128**	S1047			S1047
